# Editorial: In tune with their environment: how plant roots cope with environmental signals

**DOI:** 10.3389/fpls.2023.1234996

**Published:** 2023-07-20

**Authors:** Hugues Nziengui, Franck Anicet Ditengou

**Affiliations:** ^1^ Department of Biology, Faculty of Sciences, Université des Sciences et Techniques de Masuku, Franceville, Gabon; ^2^ Lighthouse Core Facility, Medical Center University of Freiburg, Albert Ludwigs University Freiburg, Freiburg, Germany; ^3^ Bio Imaging Core Light Microscopy (BiMiC), Institute for Disease Modeling and Targeted Medicine (IMITATE), Medical Center University of Freiburg, Albert Ludwigs University Freiburg, Freiburg, Germany; ^4^ Faculty of Biology, Institute of Biology II, Albert Ludwigs University Freiburg, Freiburg, Germany

**Keywords:** auxin, fine root, melatonin, nematode, root, overwintering

Plants constantly face fluctuating environmental conditions and their genome plasticity allows them to survive in difficult environments and flourish in favorable ones. The root system is crucial to the growth process because it anchors the plant in the soil, absorbs water and nutrients, and interacts with a variety of beneficial and harmful microorganisms. The composition and structure of soil play a crucial role in determining the survival, growth, and development of plants. This is achieved through the activation of molecular signaling pathways, which involve hormones such as auxin and their interactions. Increasing our understanding of how plant roots adapt to changing environmental conditions is essential for developing new strategies to efficiently cultivate and breed crops, as well as other plant species, including trees, that are crucial for human use and the environment.

Fine roots, with a diameter of less than 2 mm, are the most active component of the root system involved in nutrient cycling, resource capture, and global biogeochemistry (Jackson et al., 1997). Fine roots are highly sensitive to changes in the soil environment. However, it is of interest to understand how they respond to alterations in soil composition in a stable forest environment. Wen et al. conducted a study to examine the relationship between fine root morphology and soil environmental properties in *Cupressus funebris* plantations situated in four different locations (low mountain, middle hill, shallow hill, and high hill areas). These plantations exhibit differences in soil properties, including total nitrogen, alkaline nitrogen, available phosphorus, and moisture content. The researchers observed significant changes in the morphology of the first- and second-order roots. Third- to fifth-order fine roots did not respond to changes in soil chemical and physical properties. Soil moisture was correlated with root diameter and root tissue density, but negatively correlated with both specific root length and area. This suggests that when soil moisture decreases, plants develop thinner and longer fine roots in order to explore a greater volume of soil. On the other hand, total soil nitrogen, alkaline nitrogen, and available phosphorus had an opposite effect on root growth. These nutrients were positively correlated with root length and surface area, and negatively with root diameter and density. Alkaline nitrogen was found to be the most critical soil factor affecting the morphology of fine roots. While soil temperature, organic carbon, porosity, and bulk density were analyzed, none of these factors were found to be significantly correlated with any of the fine root traits.

Abril-Urias et al. discussed the transcriptional regulation of auxin-responsive genes by root-knot (RKN) and cyst (CN) nematodes. Nematodes are slender, roundworms that parasitize a wide range of crops. Auxin is a key plant hormone that plays a critical role in regulating the growth and development of roots, and the development of an auxin maximum at feeding sites is a hallmark of nematode root infection (Oosterbeek et al., 2021). The authors report that RKN induce the expression of auxin-responsive genes, such as *IAA19*, *IAA29*, and *GH3.17*. In contrast, CN repress the expression of these genes and stimulate those involved in stress and defense responses. Other genes that are commonly regulated by both types of nematodes include several members of the *AUX/IAA* gene family. This variety of auxin signaling pathways may account for the contrasting discrepancies between the transcriptional responses regulated by auxin at the CN and RKN feeding sites, at least in the early to medium stages of infection. Furthermore, both types of nematodes stimulate the expression of defense and stress response genes. This could potentially impact root growth by altering the plant’s ability to respond to environmental stresses. Nematodes that parasitize plants pose a significant challenge in agriculture and result in billions of dollars in economic losses worldwide each year. The identification of specific genes that are differentially regulated by root-knot and cyst nematodes could be valuable in developing new crop varieties that are resistant to these pests by manipulating the expression of these genes.

Both vertebrates and plants, including various species such as rice, tomato, grapevine, and tobacco, produce melatonin (Dubbels et al., 1995; Hattori et al., 1995). To gain insight into the function of melatonin in plants, Wei et al. conducted a time-resolved transcriptome experiment to analyze the relationship between melatonin and auxin, two tryptophan derivatives, in soybean. They found that both compounds regulate similar sets of genes. Exogenously applied melatonin activates auxin biosynthesis and response genes. By enhancing the expression of *YUCCA* genes, it is presumed that the levels of auxin are increased, which in turn modulates the genes responsible for auxin transport (*AUX/LAX* and *PIN/PIL*) and response. Similar to auxin, melatonin induces the expression of auxin receptor genes such as *TIR1*, *AFB3*, and *AFB5*, as well as other auxin-responsive genes, including *IAA*, *ARF*, *GH3*, and *SAUR*-like genes. The stimulatory effects of low concentrations of melatonin on auxin biosynthesis and signaling genes offer new possibilities for improving crop yields. Further research is necessary to establish the ideal concentration and application techniques of melatonin for diverse crop cultivars and species, considering different environmental conditions.

During prolonged exposure to cold temperatures in winter, the aboveground portion of winter turnip rape begins to wither at the eight-to-nine-true leaf stages. However, the root collars (including the shoot apical meristem) are able to survive and overwinter. Liu et al. offer valuable insights into the genetic mechanisms underlying root development and transcriptional memory during overwintering in *Brassica rapa* L. cultivated in the field. By utilizing winter rapeseed, the only overwintering cruciferous oilseed crop in northern China, researchers discovered differentially expressed genes involved in various cellular pathways, such as phenylpropanoid biosynthesis, plant hormone signal transduction, and MAPK signaling. The identification of candidate genes involved in overwintering memory and cold resistance provides valuable genetic resources for breeding cold-resistant varieties of *Brassica rapa* L. and other crops. These varieties have the potential to increase crop yields and improve food security in regions with harsh winter conditions.

These original research articles report on relevant aspects of plant adaptation to changing soil and environmental conditions. They describe how molecular pathways involving auxin production and signaling, in relation to other metabolites such as melatonin, modulate plant adaptation to various ecosystems. In doing so, these studies enhance our understanding of root physiology and its mechanisms for dealing with abiotic and biotic stresses. Several genes involved in diverse signaling pathways have been identified in these studies. Characterizing these genes is a crucial step in understanding how plants adapt to changes in soil composition. This process can aid in producing crop cultivars and species that are better suited to specific habitats.

## Author contributions

All authors listed have made a substantial, direct, and intellectual contribution to the work and approved it for publication.
